# Dislocation-Assisted Quasi-Two-Dimensional Semiconducting Nanochannels Embedded in Perovskite Thin Films

**DOI:** 10.1021/acs.nanolett.2c03404

**Published:** 2023-06-12

**Authors:** Huaixun Huyan, Zhe Wang, Linze Li, Xingxu Yan, Yi Zhang, Colin Heikes, Darrell G. Schlom, Ruqian Wu, Xiaoqing Pan

**Affiliations:** † Department of Materials Science and Engineering, University of California−Irvine, Irvine, California 92697, United States; ‡ Department of Physics and Astronomy, University of California−Irvine, Irvine, California 92697, United States; § State Key Laboratory of Surface Physics, Key Laboratory of Computational Physical Sciences and Department of Physics, Fudan University, Shanghai 200433, China; ∥ Irvine Materials Research Institute, University of California−Irvine, Irvine, California 92697, United States; ⊥ Department of Materials Science and Engineering, Cornell University, Ithaca, New York 14850, United States

**Keywords:** dislocation, nanochannel, perovskite thin film, Schottky junction, defect engineering

## Abstract

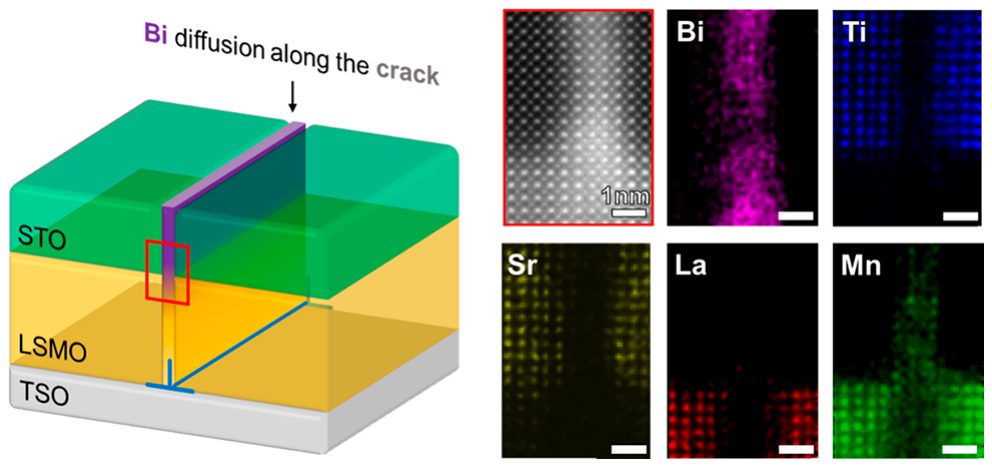

Defect engineering in perovskite thin films has attracted extensive attention recently due to the films’ atomic-scale modification, allowing for remarkable flexibility to design novel nanostructures for next generation nanodevices. However, the defect-assisted three-dimensional nanostructures in thin film matrices usually has large misfit strain and thus causes unstable thin film structures. In contrast, defect-assisted one- or two-dimensional nanostructures embedded in thin films can sustain large misfit strains without relaxation, which make them suitable for defect engineering in perovskite thin films. Here, we reported the fabrication and characterization of edge-type misfit dislocation-assisted two-dimensional BiMnO_*x*_ nanochannels embedded in SrTiO_3_/La_0.7_Sr_0.3_MnO_3_/TbScO_3_ perovskite thin films. The nanochannels are epitaxially grown from the surrounding films without noticeable misfit strain. Diode-like current rectification was spatially observed at nanochannels due to the formation of Schottky junctions between BiMnO_x_ nanochannels and conducting La_0.7_Sr_0.3_MnO_3_ thin films. Such atomically scaled heterostructures constitute more flexible ultimate functional units for nanoscale electronic devices.

Epitaxial solid thin films and heterostructures may manifest various unusual properties because of the interplay of many degrees of freedom at the interfaces. They offer high uniformity, low current leakage, and high reliability for the design of diverse nanodevices, outperforming amorphous and polycrystalline films. Furthermore, their properties such as dielectric constant, piezoelectric displacement, and ferroelectric polarization can be tuned in a large range by changing growth directions, lattice strain, and substrate. However, one major limitation of crystalline film growth is that once the substrate or the growth orientation is set, further modification to control or alter the thin film structures becomes difficult. One promising solution is to introduce defects in thin films, which can have a remarkable impact on thin film growth dynamics. Indeed, commonly observed defects such as dislocations, vacancies, and impurities play an important role in epitaxies of films and may induce unique local nanostructures with novel properties.^1−8^ For instance, impurity defects can induce the formation of nanopillar-embedded thin films, which show excellent properties such as enhanced conductivity,^9^ photovoltaic response,^10^ ferroelectricity,^11^ and magnetoelectricity.^12,13^ These films offer promise for the development of multifunctional nanodevices. Similarly, dislocations can pin the local atomic structures and assist the formation of arrays of one-dimensional (1D) nanochannels in two-dimensional (2D) or three-dimensional (3D) materials.^14−16^ These dislocation-assisted lower-dimensional nanostructures embedded in higher-dimensional crystalline matrices can sustain large strain and thus offer a good control over local electronic structure.^16^ Such routes open a path to fabricate stable nanostructures with refined patterns and allow the design of nanodevices with high flexibility and integrability. Specifically, a combination of different phases in a single material system provides a coupling effect, *e.g.*, metallic-nanopillar^17^ and nanopillar-ceramic^18^ coupling, and can form novel interfaces such as p–n junctions.^19,20^ The further development of the capability of producing ordered patterns of dislocation-assisted 2D nanochannels into 3D epitaxial thin films is obviously rewarding.

In this work, we demonstrate the fabrication of arrays of dislocations in a controlled manner during thin film growth, and use these dislocations to form ∼1 nm wide 2D nanochannels in 3D SrTiO_3_ (STO)/La_0.7_Sr_0.3_MnO_3_ (LSMO)/TbScO_3_ (TSO) perovskite thin films. As illustrated in Figure 1a, 25 unit cells of STO thin film were grown on 50 unit cells of LSMO on single crystal (110)_O_ (subscripts O denote orthorhombic indices) TSO substrate by reactive-molecular beam epitaxy (MBE). A bismuth flux was then supplied to 100 nm bismuth equivalent growth time to deposit a bismuth layer on the STO film. The atomic structures and compositions were characterized using cross-sectional scanning transmission electron microscopy (STEM) and energy dispersive X-ray spectroscopy (EDS), respectively. The electric properties and electronic structures of interfaces between the nanochannels and the LSMO films were studied using conductive atomic force microscopy (c-AFM), electron energy loss spectroscopy (EELS) and the first-principles calculations, indicating a formation of Schottky junctions.

**Figure 1:**
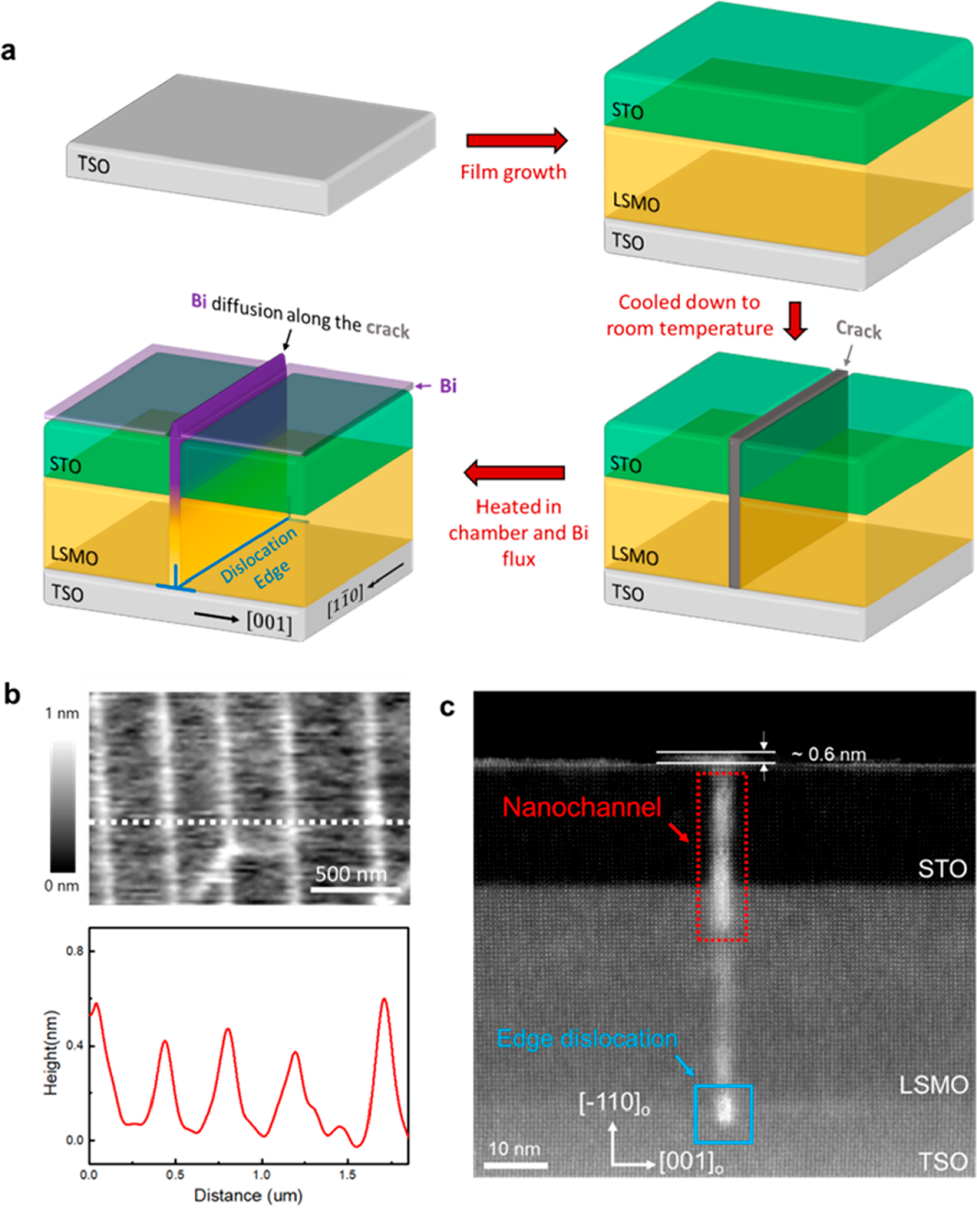
Nanochannel fabrication process and its characterizations. (a) Schematic illustration of the fabrication process of the nanochannel. (b) AFM surface topology measurement and corresponding line scan along the plane TSO (−110)_O_, and (c) cross-sectional HAADF STEM image. A dislocation is observed at the LSMO/TSO interface and right below the nanochannel.

Figure 1a illustrates the concept of the fabrication process for the nanochannels. The 10 nm STO and 20 nm LSMO films were first grown on TSO substrate and cooled down to room temperature. Due to the different lattice parameters and thermal expansion coefficients between LSMO and TSO films, the strain in LSMO is 1.16% at 650 °C and increased to 1.34% at room temperature (Table S1). After cooling, cracks began to form from the dislocations as the large strain induced by the lattice mismatch between LSMO^21^ and TSO^22^ at room temperature could not be relaxed by the creation of dislocations only.^23^ Subsequently, the STO/LSMO/TSO thin films were heated up to 650 °C in the chamber again, resulting in cracks recovering and chemical elements diffusion. At the same time, Bi flux was also kept on, depositing Bi not only on top of STO film but also into the recovering cracks. As such, rather than fully growing back to uniform STO/LSMO films, top part of the cracks formed quasi-2D nanochannels, which consists of Bi compounds, along the extra half plane of the dislocation (Figure S1).

The surface topography of the Bicoated thin film along the TSO (110)_O_ plane was measured using AFM as shown in Figure 1b, giving white lines distributed across the film. The corresponding topographic line scan, indicated by the white dashed line, shows several bumps about 0.7 nm higher than the film surface. In order to study the structural information under the surface, we prepared a cross-sectional TEM sample and performed a cross-sectional high angle annular dark field (HAADF) STEM imaging through the in-plane direction (see details in the Supporting Information). Figure 1c displays an overview of STO/LSMO/TSO thin films along the TSO [11̅0]_O_ direction. The red dotted rectangle marks a nanochannel with ∼1 nm (2–3 u.c.) in dimension, which penetrates through the STO film and terminates in the LSMO film. The blue square indicates a dislocation at the STO/LSMO interface right below the nanochannel. Corresponding Geometric Phase Analysis (GPA) maps (Figure S2) show a typical dislocation strain loop,^24,25^ further confirming the existence of the dislocation at LSMO/TSO interface. A tiny bump is also observed at the top of the nanochannel with a height of ∼0.6 nm and the EDS measurement (Figure S3) indicates that it is Birich. This is consistent with the AFM topography mapping and confirms that the white lines in the AFM image are from the nanochannels. By combining the structure information from top and cross-sectional views, a three-dimensional nanochannel structure is schematically reconstructed (Figure 1a), showing that the nanochannel forms exactly along the extra half plane of the edge dislocation as Bi atoms diffuse into the crack.

Atomic-resolution EDS was then conducted on the nanochannel area to characterize the atomic structure and chemical composition in details. Interestingly, a close-up HAADF STEM image gives a clear perovskite atomic structure of the channel, and corresponding atomic-resolution EDS results (Figure 2b–f) reveal that the most significant signal detected in the nanochannel is Bi, Mn, and O with an overall ratio of ∼1:1:2.44, indicating a formation of BiMnO_x_ (BMO). The signal of Ti and Sr is very weak with elemental compositions of 0.5% and 0.8%, respectively, meaning that they are not preferred in the nanochannel. The weaker oxygen signal in the nanochannel region than that of the matrix materials (STO and LSMO) is likely due to oxygen vacancies. The atomic-resolution EDS data collected from a pure STO/LSMO interface (Figure S4) show that the interface is atomically sharp, ruling out the possible element diffusions.

**Figure 2:**
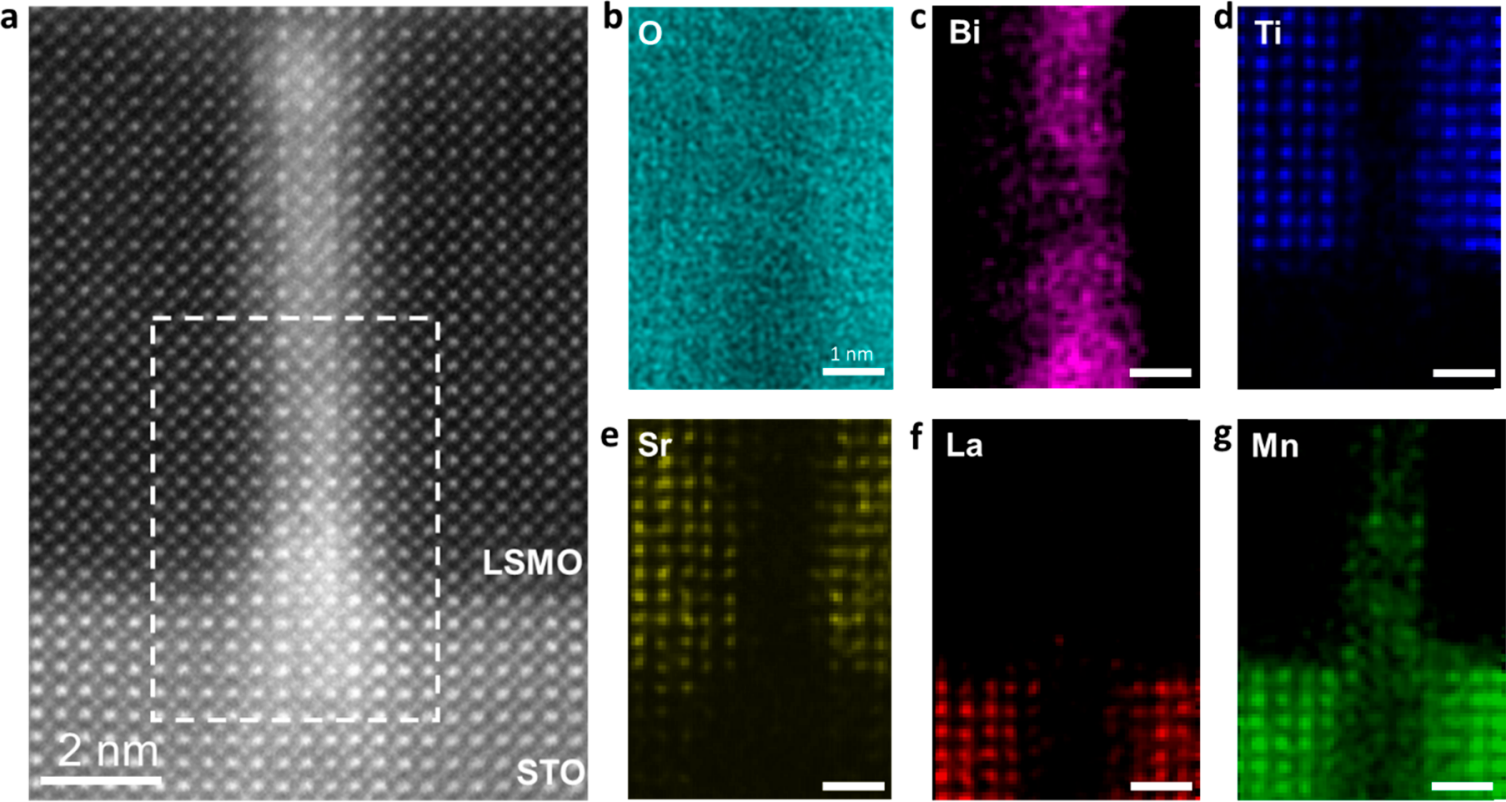
Nanochannel atomic-resolution EDS maps. (a) Cross-sectional HAADF STEM image. (b–g) Atomic-resolution EDS maps of (b) O, (c) Bi, (d) Ti, (e) Sr, (f) La, and (g) Mn collected from the white dashed box in a, revealing an atomically sharp nanochannel.

As the film surface topography has been resolved, a c-AFM conductivity measurement was conducted to further analyze the electronic properties of the nanochannels. 6 V and −6 V bias were applied between the top surface and LSMO layer of the film, respectively, as shown in Figure 3a. The conductivity mapping indicates that the nanochannels have an enhanced conductance as a positive bias is applied but becomes insulating when a negative bias is applied. A corresponding line scan (Figure 3b) was performed along the white dashed line in Figure 3a, showing that the current signal increases to a value up to ∼7 pA only at the nanochannels. One representative current–voltage (*I*–*V*) curve at a single nanochannel shows ∼10 pA at 6 V and almost zero current at −6 V (Figure 3d), revealing a rectifying diode characteristic. The rectification ratio (forward current/reverse current) of the nanochannel is as high as 90 at ±6 V, which is comparable to that measured from other common systems such as Si/MoS_2_,^26^ WS_2_/MoS_2_,^26^ and Au/n-ZnO.^27^ In contrast, the *I*–*V* curve measured from STO film gives no meaningful signal because STO is a typical insulator. Such conductive behavior suggests a possible formation of Schottky junctions, which usually exist at metal and semiconductor interfaces, in-between the nanochannels and LSMO films as indicated by the red dashed boxes in the schematic drawn in Figure 3c. Since LSMO has been reported as a half metal,^28,29^ the nanochannels, mainly BMO with oxygen defects, should behave as an n-type semiconductor. Previous study has also confirmed that BMO with oxygen vacancies show n-type conduction due to the vacancy-induced electron donors.^30^

**Figure 3:**
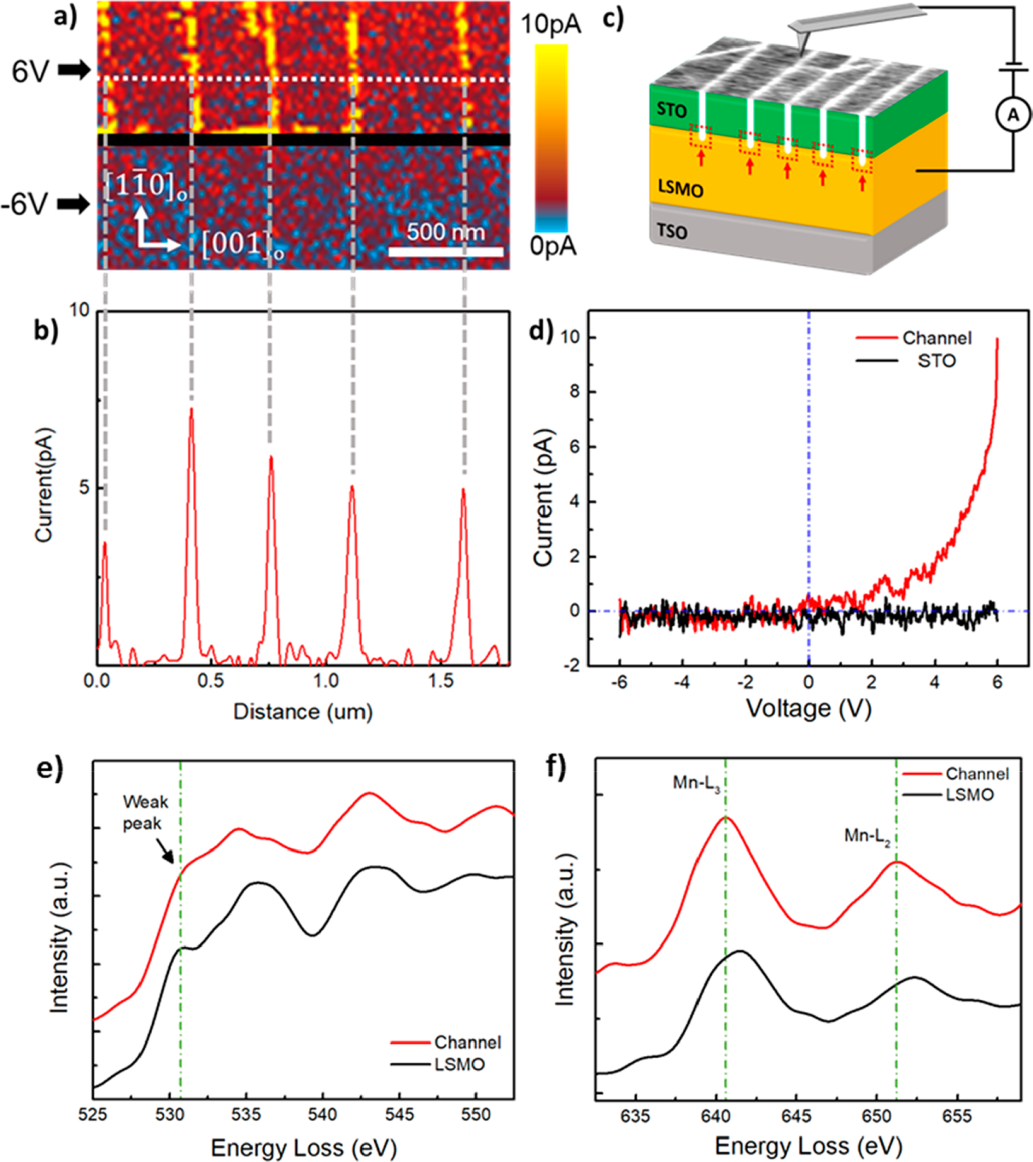
Conductivity measurement and EELS characterizations of the nanochannel and LSMO films. (a) C-AFM conductivity mapping of the film surface. (b) A current line-scan orthogonal to the channels. The scanning path is indicated by the white dashed line in a. (c) A scheme of the c-AFM measurement set up. The white lines and red dashed boxes indicate nanochannels and junctions between nanochannels and LSMO films, respectively. (d) *I*–*V* curves measured on the channel and on the STO film. (e) Oxygen K-edge of the nanochannel (red) and the LSMO films (black). (f) Manganese L_2_- and L_3_-edges of the nanochannel (red) and the LSMO films (black).

Investigating the near edge structure of core-loss EELS of oxygen and B-site Mn can help us to determine the valence states and therefore the BMO electrical conductivity type (p- or n-type). Spatial dependent EELS of O-K and Mn-L_2,3_ edges were acquired from the regions in the channel and the LSMO film, as shown in Figure 3e and f. The O–K edge from the LSMO is consistent with the LSMO bulk data previously reported.^31,32^ The O–K edge from the channel, however, is different because its first subpeak at 531 eV almost disappears. The first subpeak of O–K edge is typically attributed to the covalent interaction between O 2p and Mn 3d orbitals.^33^ A weakening of this peak means less Mn and O interaction and lower Mn oxidation state due to oxygen vacancies.^32,34^ This is further confirmed by the peak onset difference comparison between O and Mn. The peak onset energy difference gives the energy separation between the inner shell energy levels of O 1s and Mn 2p, ruling out the need for the precise energy calibration.^35^ As a rule of thumb, less energy difference indicates a lower Mn oxidation state. The peak onset differences between the O-K edge and Mn-L_3_ edge are 110 eV for LSMO and 108.1 eV for the nanochannel,^36^ revealing a lower Mn oxidation state in the BMO nanochannel. The two methods, together with the oxygen EDS map, therefore clearly indicate the existence of oxygen vacancies in the nanochannel, resulting in an n-type feature as we hypothesized.

Now, questions may raise as to why an almost pure BMO perovskite structure forms in the nanochannel in such a complex growth condition. In order to investigate the energetics of the growth and to determine the Schottky barrier height between BMO nanochannel and the LSMO film, density functional theory (DFT) calculations were performed for several model systems. We considered all the possible compounds that can be formed in the nanochannel, including perovskite-type La_1–*x*_Bi_*x*_MnO_3_, Sr_1–*x*_Bi_*x*_MnO_3_, and Sr_1–*x*_Bi_*x*_TiO_3_. Their phases were set to be ferromagnetic with space group *Pbnm* as shown in Figure S6a, which is the ground state of BiMnO_3_ under the lattice matching restriction in the nanochannel (Table S2). The final product is the compound that minimizes the Gibbs free energy Δ_r_*G*, defined as
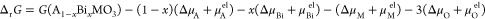
1where *A* represents La or Sr, M denotes Ti or Mn, *G* is the Gibbs free energy of the corresponding compound, and Δμ_α_ is the chemical potential of element α with respect to its elemental phase μ_α_^el^ (oxygen molecule for O, and solid phase for others). Here, μ_α_^el^ is set to be equal to the DFT total energy, while Δμ_α_ is related to experimental synthesis conditions. Using the tabulated^39^ values of enthalpy, entropy and heat capacity for O_2_, we yielded Δμ_O_ = −1.00 eV with respect to 1/2*E*_DFT_ (O_2_) at the experimental conditions of *T* = 650 °C and *P* = 1 bar (see details in the Supporting Information). For materials in solid or liquid phase, we neglected the enthalpy and entropy contributions, and simply used Δμ_Bi_ = 0 and *G*(A_1–*x*_ Bi_*x*_MO_3_) = *E*_DFT_(A_1–*x*_Bi_*x*_MO_3_). This approximation leads to a maximum error of about 0.1 eV/atom according to our estimates (Table S3), which does not change our main discussions. The chemical potentials of other elements, La, Sr, Mn and Ti, are restricted by their reservoirs, LSMO and STO. Using various thermodynamic stability conditions (see details in the Supporting Information), we deduced that −8.66 eV ≤ Δμ_La_ ≤ – 8.01 eV, – 6.59 eV ≤ Δμ_Sr_ ≤ – 5.42 eV, – 4.73 eV ≤ Δμ_Mn_ ≤ – 4.08 eV when LSMO acts as the reservoir (Figure 4a), and −6.42 eV ≤ Δμ_Sr_ ≤ – 5.09 eV, – 9.75 eV ≤ Δμ_Ti_ ≤ – 8.42 eV when STO acts as the reservoir. Then the stability of all possible products in the nanochannel can be compared using eq 1. The calculated reaction Gibbs free energy Δ_r_*G* is displayed in Figure 4b and Figure S6b, in which the green shaded area represents the allowed chemical potential range for La and Sr. Clearly, BMO has the lowest Δ_r_*G* compared to that of La_1–*x*_Bi_*x*_MnO_3_ and Sr_1–*x*_Bi_*x*_MnO_3_, indicating Mn elements can be extracted from LSMO and form BMO in the nanochannel. In contrast, STO has the lowest Δ_r_*G* compared to Sr_1–*x*_Bi_*x*_TiO_3_, suggesting Ti atoms stably resides in the STO films and do not participate in the rearrangement during the growth. Despite the presence of defects in the sample, these DFT results from crystalline models align well with the EDS maps (Figure 2) and explain the tendency of upward Mn diffusion.

**Figure 4:**
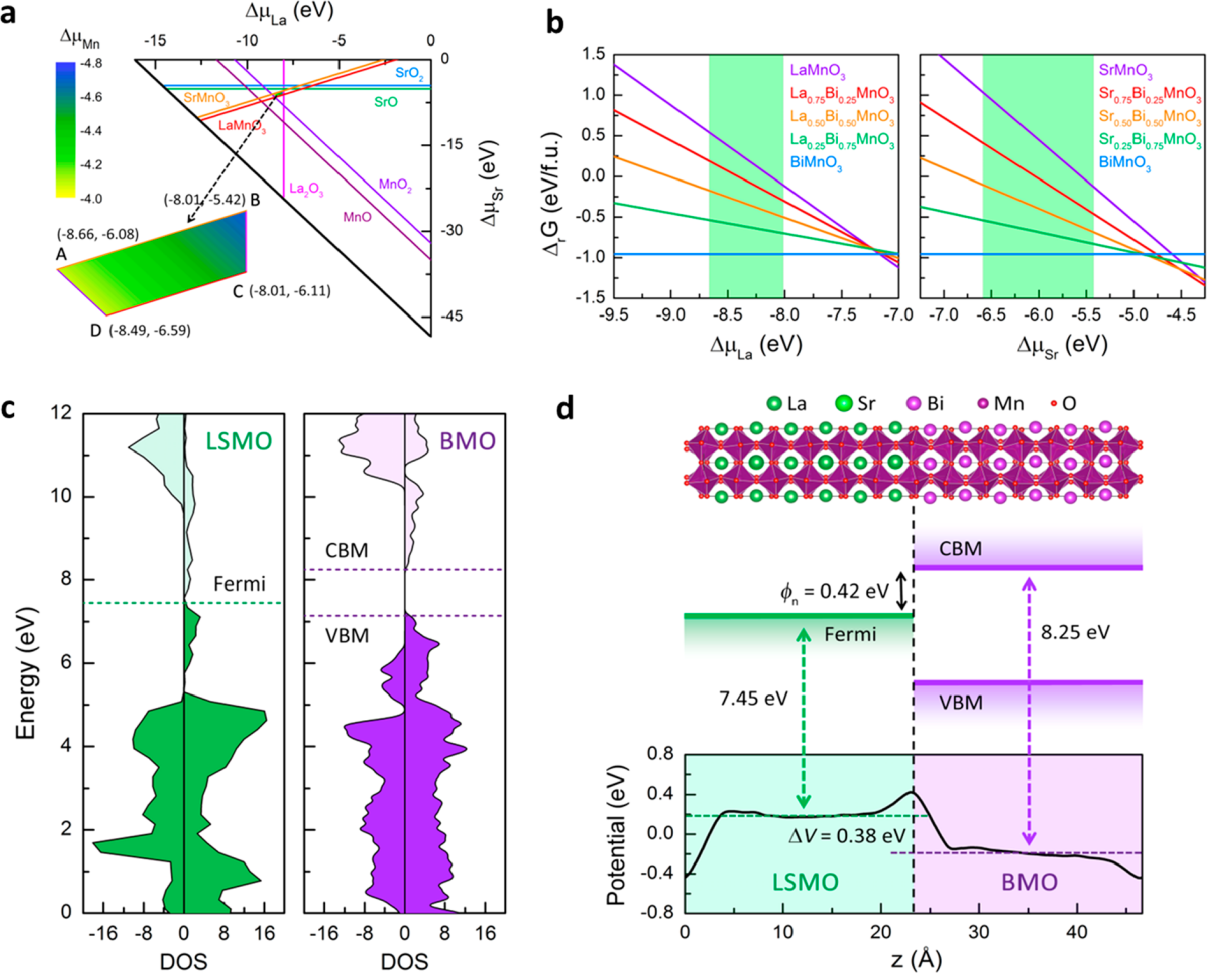
Formation mechanism of BiMnO_x_ nanochannels and the Schottky barrier. (a) Calculated chemical potential diagram for LSMO at *T* = 650 °C and *P* = 1 bar (Δμ_O_ = −1.00 eV). Each line corresponds to one of the constraint formulas of eq 5 in the Supporting Information. The color filled polygon (marked by points A, B, C, and D) shows the range of Δμ_La_ and Δμ_Sr_ in which LSMO is stable. The numbers at each point denote the corresponding exact values of (Δμ_La_, Δμ_Sr_) in unit of eV. For a given point in the polygon, Δμ_Mn_ can be determined via eq 4 in the Supporting Information, and is visualized by color coding. (b) The reaction Gibbs free energy Δ_r_*G* as a function of Δμ_La_ and Δμ_Sr_ for La_1–*x*_Bi_*x*_MnO_3_ and Sr_1–*x*_Bi_*x*_MnO_3_, respectively. The green shaded area represents the allowed chemical potential range as determined in (a). Here we adopted an intermediate value of −4.40 eV for Δμ_Mn_. The conclusions do not change for other values of Δμ_Mn_ according to eq 1. (c) Density of states (DOS) of bulk LSMO and perovskite-type BMO, calculated by using the HSE06 hybrid functional^37^ to get a more accurate description on the conduction band minimum (CBM) and valence band maximum (VBM). The average electrostatic potential is set to 0 eV. The lattice constants of BMO are fixed to that of LSMO to simulate the restriction of lattice matching. (d) Top panel: The supercell model used to simulate LSMO/BMO junctions. Middle panel: Schematic diagram of the band alignments at LSMO/BMO interface. Bottom panel: The macroscopic average of the electrostatic potential for electrons of the LSMO/BMO supercell, calculated by using the macroscopic average technique.^38^

To explain the observed diode-like current rectification at the BMO/LSMO interface, we calculated their electronic structures. As shown in Figure 4c, bulk LSMO is a half-metal,^40^ with a Fermi level of *E*_Fermi_^LSMO^ = 7.45 eV with respect to its average electrostatic potential. While bulk perovskite-type BMO is a semiconductor, with the conduction band minimum (*E*_CBM_^BMO^) lies 8.25 eV above its average electrostatic potential. At the LSMO/BMO junction, a potential difference of Δ*V* = 0.38 eV is built across the interface (Figure 4d). A Schottky barrier hence forms between LSMO and the n-type BMO as schematically illustrated in Figure 4d. The Schottky barrier height (SBH) can be determined using
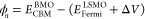
2and a considerable SBH of 0.42 eV is produced between the LSMO and n-type BMO. While acknowledging the differences between perfect crystals here and defective and rough interfaces in experiment, our theoretical model provides an explanation for the observed rectifying diode behavior at a qualitative level, which was previously discussed.

Defect engineering is a very important and widely used method for manipulating perovskite thin film structures at the atomic scale. It enables remarkable flexibility for designing nanodevices and often leads to unexpected structures and properties. In this work, we utilized edge-type misfit dislocations, which induced by LSMO/TSO lattice mismatch, to create the cracking during the thin film growth, and eventually assist the epitaxial formation of BMO nanochannels. Furthermore, the nanochannels are formed along the dislocation extra half-plane with a width of ∼1 nm, confirming the high accuracy of the dislocation-guided process. Such controllable quasi-2D nanochannels embedded in 3D STO/LSMO films offers mixed-dimensional modifications and excellent flexibility.

A recent work from Yeo et al. reported a conductive cracked wall in STO/DyScO_3_^4^ The conduction is induced by STO with oxygen vacancies. In our case, however, the EELS results (Figure S5) collected from near-crack STO regions show the same Ti and O edges as the bulk STO results previously reported,^41^ confirming the STO near BiMnO_x_ nanochannel region is free of oxygen vacancy and thus ruling out the effect of surrounding STO on BiMnO_x_ nanochannel conductance. On the other hand, our EDS, EELS, and simulation results have confirmed the BiMnO_x_ channel is oxygen deficient, confirming the conduction is induced by the BiMnO_x_ nanochannel.

It is notable that bulk phases of BMO in nature are triclinic, with the *C*2 or *C*2/*c* structure.^42−44^ Based on the HAADF STEM images and DFT calculations, however, BMO observed in the nanochannels shows orthorhombic *Pbnm* structure (Table S2), which is close to that of the matrix material STO. Such structure change is guided by the matrix materials in order to lower system energy with the lattice match. The GPA maps (Figure S2) show no obvious strain at the BMO/STO or BMO/LSMO interface, confirming the negligible structural deviation from BMO to the matrix materials and good strain tolerance of BMO as well. By using this fabrication method, we can potentially grow many perovskite materials with different atomic structures into the nanochannel and manipulate the Schottky barrier and rectification properties.

The use of c-AFM in this work allows numbers of parameters, such as conductivity and surface roughness, to be explored on the thin film surface. The technique usually offers very high spatial resolution down to subnanometers.^45^ At such scale, the local defect patterns and defect induced local microscopic structures near surface can be unambiguously determined. However, AFM only measures the surface region, the microscopic structures beneath the surface are still missing. This is complemented by the advanced TEM techniques which have sub-ångström spatial resolution capability and enable the probe of local defect patterns and related properties of surface and interior regions of materials. In the present study, we used atomic-resolution HAADF-STEM to characterize the structures of the quasi-2D nanochannels and to resolve the corresponding defect locations; atomic-resolution EDS to check the nanochannel chemical compositions; and EELS to confirm the existence of oxygen vacancies. The information provided by these advanced TEM techniques helps us to draw a conclusion that the nanochannel is almost purely oxygen-deficient BMO, and Schottky junctions are formed between BMO nanochannels and LSMO films. The use a suite of techniques is powerful for the defect engineering of advanced materials and the present study is a good showcase.

In conclusion, we have fabricated and characterized an array of dislocation-assisted quasi-2D nanochannels that embedded in STO film. Atomic-resolution HAADF STEM and EDS results show the nanochannels are oxygen-deficient BMO. EELS data further confirms that there are oxygen vacancies exist in the nanochannels, accompanying with a reduced oxidation state of Mn and the formation of an n-type material in the channel. As a result, the *I*–*V* curve of the channel shows a rectifying characteristic, indicating the formations of Schottky junctions between the nanochannels and LSMO films. This work demonstrates the possibility of creating ultrathin 2D conductive path in 3D epitaxial perovskite films, giving rise to novel and flexible multidimensional manipulations for multifunctional oxides. We anticipate that our results will inspire subsequent studies in this realm and will lead to new approaches of microstructural engineering that is needed for the design of diverse nanodevices.

## Methods

### Thin Film Growth and TEM Specimen Preparation

The 25 unit cell thick SrTiO_3_ (STO) thin films were grown on 50 unit cell La_0.7_Sr_0.3_MnO_3_ (LSMO) on single crystal (110)_O_ TbScO_3_ (TSO) substrate by reactive-molecular beam epitaxy (MBE). Then the Bi flux was kept on for 100 nm Bi equivalent growth time to deposit a Bi layer on top of SrTiO_3_ films in the ultrahigh vacuum deposition system. TEM specimens were prepared by mechanical polishing followed by argon ion milling using Gatan PIPS II.

### Conductive-AFM Setup

AFM and c-AFM measurements were performed using a commercial Asylum Research MFP-infinity system. Nanosensors PPP–EFM cantilever were used in this study. Conductive platinum-coated tip was used for the c-AFM measurements in Figure 3. In order to eliminate the effect of platinum tip/BMO junction, a diamond-coated tip was used, and a similar *I*–*V* was acquired as shown in Figure S7. The c-AFM images were acquired with a bias voltage *V*_appl_ = ± 6 V between the tip (grounded) and the sample.

## Supporting Information Available

Detailed description of lattice mismatch calculations, nanochannel GPA analysis, STO EDS and EELS results, additional c-AFM measurements, additional DFT calculations, and TEM imaging techniques (PDF)
