# Supplementary material for: Dislocation-Assisted Quasi-Two-Dimensional Semiconducting Nanochannels Embedded in Perovskite Thin Films
Source: Nano Lett. 2023 Jun 12;23(12):5409–16. doi: 10.1021/acs.nanolett.2c03404 (PMC10311590; doi:10.1021/acs.nanolett.2c03404)
Supplement: Supplementary file 1 — nl2c03404_si_001.pdf [file nl2c03404_si_001.pdf]

Supporting Information

Dislocation-assisted Quasi-Two-dimensional Semiconducting Nanochannels Embedded in Perovskite Thin Films

Huaixun Huyan[1], Zhe Wang[2, 3], Linze Li[1], Xingxu Yan[1,4], Yi Zhang[1], Colin Heikes[5], Darrell G. Schlom[5], Ruqian Wu[2], Xiaoqing Pan[1,2,4*]*

[1.] Department of Materials Science and Engineering, University of California – Irvine, Irvine, CA 92697, USA
[2.] Department of Physics and Astronomy, University of California – Irvine, Irvine, CA 92697, USA
[3.] State Key Laboratory of Surface Physics, Key Laboratory of Computational Physical Sciences and Department of Physics, Fudan University, Shanghai 200433, China
[4.] Irvine Materials Research Institute, University of California – Irvine, Irvine, CA 92697, USA
[5.] Department of Materials Science and Engineering, Cornell University, Ithaca, NY, 14850 USA
* Corresponding author: xiaoqingp@uci.edu; wur@uci.edu

Table S1. Lattice mismatch between the LSMO film and TSO substrate at room temperature (RT) and at 650 ℃. The latter is calculated using the thermal expansion coefficients of the LSMO[1] and TSO[2].

Compound	Lattice parameter Å	Thermal expansion coefficient /K	LSMO mismatch stain (%) at RT	LSMO mismatch stain (%) at 650 °C
LSMO	7.809	1.28 x 10^{-5}	0	0
TSO	7.915	1.01 x 10^{-5}	1.34	1.16

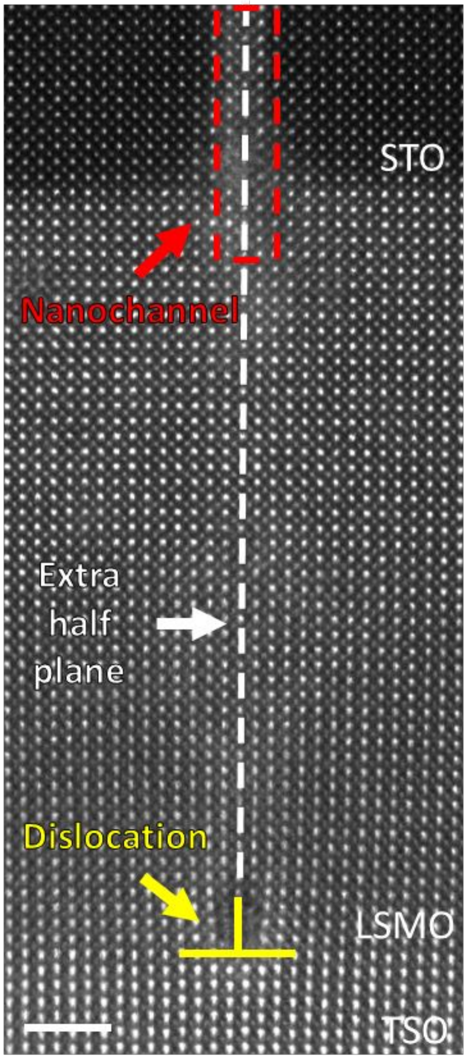

Figure S1. A close-up HAADF STEM image shows that a nanochannel is formed along the dislocation extra half plane. Scale bar, 2 nm.

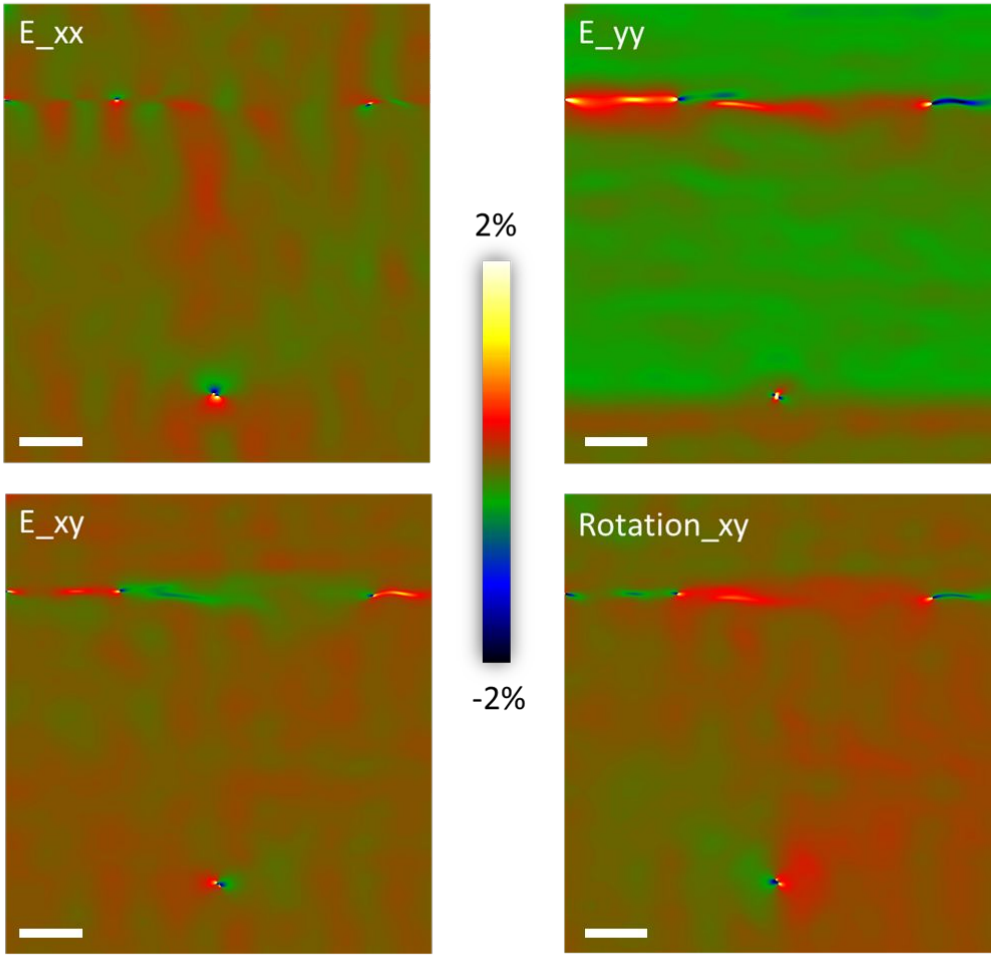

Figure S2. Corresponding geometric phase analysis (GPA) of the HAADF STEM image shown in figure 1, revealing dislocation induced strain at the LSMO/TSO interface. Note the contrast at the surface region is artifact due to the different atomic structures (vacuum/STO). The striped patterns are caused by scanning noise in the STEM image. Scale bar, 10 nm.

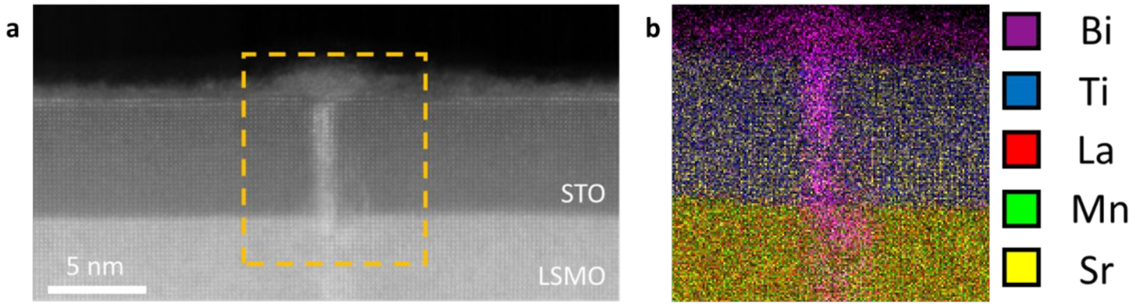

Figure S3. a) HAADF STEM image of a nanochannel and b) corresponding EDS mapping, indicating the bump is Bi-rich.

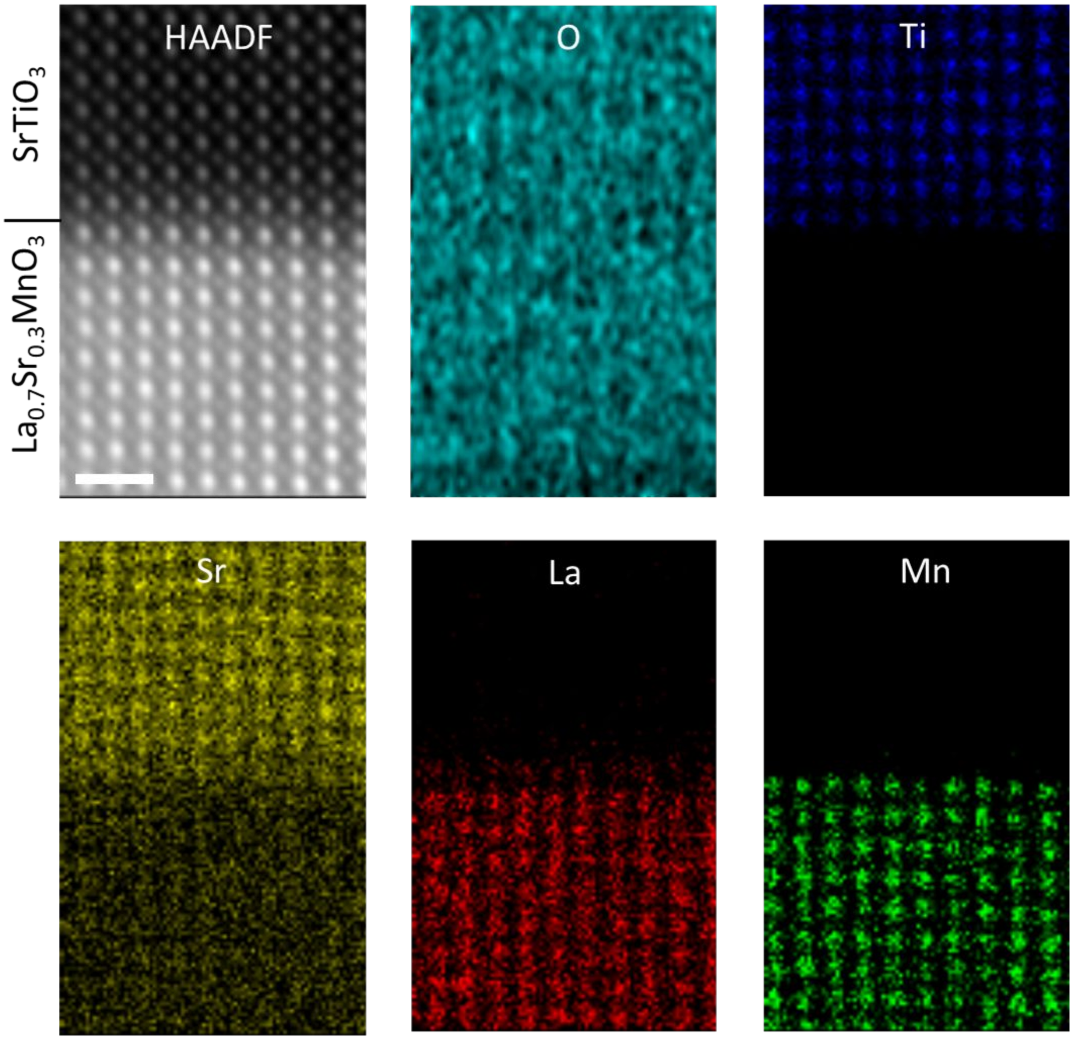

Figure S4. Chemical composition across the STO/LSMO interface. The HAADF STEM image at left is simultaneously captured with EDS maps. The atomic scale STEM-EDS images measured for each element show an atomically sharp STO/LSMO interface. Scale bar, 1 nm.

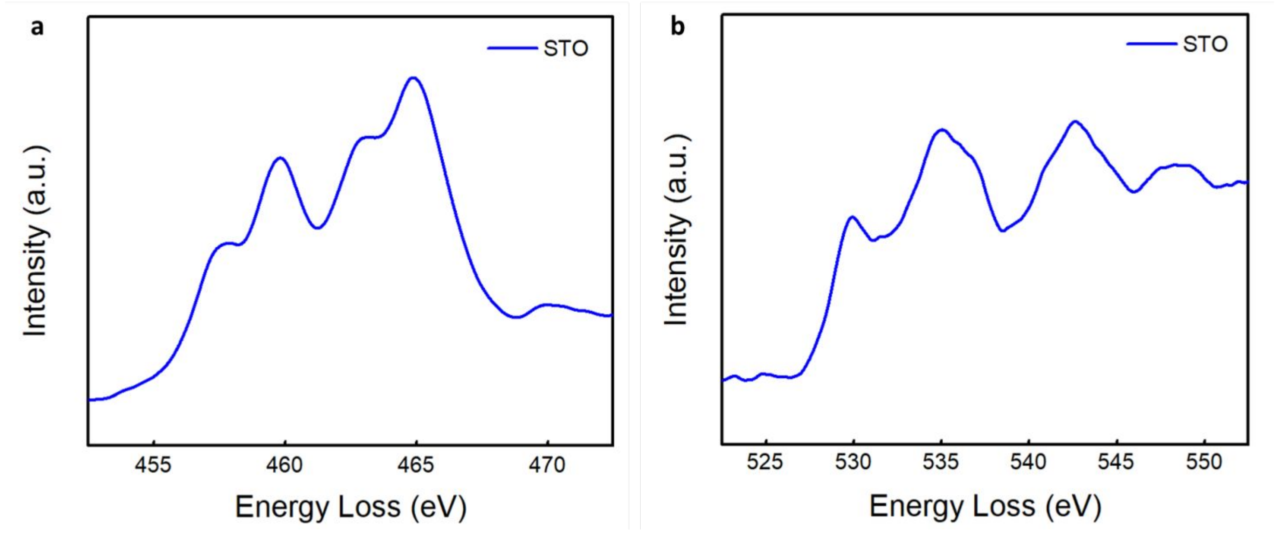

Figure S5. EELS peaks of STO films. a) Ti L-edge and b) Oxygen K-edge of the STO films. The Ti and O peaks are consistent with the STO bulk data previously reported[3].

Table S2. Calculated total energies of BMO for all the five possible perovskite-type structures, with four possible magnetic structures (FM, A-AFM, C-AFM and G-AFM) for each of them. In nature, bulk BMO crystallizes in a triclinic structure in *C2* or *C*2/*c* space group[4-6], with a ferromagnetic ground state below its Curie temperature of 99~105 K[5] and a band gap of ~1.1 eV[7]. However, the restriction of lattice matching forces BMO to adopt a perovskite-type structure in the nanochannels. To simulate such a restriction, all the lattice constants are fixed to that of LSMO in our calculations. Depending on the rotation of MnO_6 octahedron, its structure may belong to space group *Pm*-3*m*, *I*4/*mcm*, *Pbnm* and *R*-3*c*. In addition, it was reported[8] that the cubic phase of BMO can be ferroelectric (space group *P*4*mm*), which was also considered in our calculations. It is clear that the ground state is FM with space group *Pbnm*.

Energy (eV/f.u.)	FM	A-AFM	C-AFM	G-AFM
Pm-3*m* (cubic)	0.765	0.911	1.112	1.344
*I*4/*mcm* (tetragonal)	0.328	0.480	0.608	0.646
Pbnm (orthorhombic)	0.000	0.068	0.123	0.068
R-3*c* (rhombohedra)	0.232	0.202	0.243	0.742
*P*4*mm* (Ferroelectric)	0.764	0.911	0.683	0.695

Table S3. Estimated error of different approximations for calculating $\Delta_f G(T, P)$ at $T = 650°C$, $P = 1$ bar, where $\Delta_f G(T, P)$ is the Gibbs free energy of formation. Approximation I: The enthalpy and entropy contributions are taken into account for gas phase only, and are neglected for solid and liquid phase. This approximation is based on the fact that heat capacity differences between solid (liquid) phases are generally quite small, leading to cancellations when calculating $\Delta_r G$. Approximation II: The enthalpy and entropy contributions are neglected for all phases, i.e., using $\Delta_f G$(0 K) to approximate$\Delta_f G(T, P)$. The data under 0 K are taken from the National Bureau of Standards tables[9], and the data of $T = 650°C$, $P = 1$ bar are deduced from the M. Binnewies and E. Mike thermodynamic tables[10]. Due to the incompleteness of the thermodynamic tables in particular for 0 K, our estimation is not able to cover all the involved system of this work, but is restricted in La_2O_3, TiO, TiO_2 (anatase), TiO_2 (rutile), Ti_2O_3 and Ti_3O_5. The numbers of $\Delta_f G(T, P)$ are given in unit of eV/atom, and the corresponding errors are listed in parentheses. Clearly, approximation I which we adopted in the main text leads to a maximum error of about 0.1 eV/atom, which does not change our conclusions.

$\Delta_f G$(T, P)	Exact value (650°C, 1 bar)	Approximation I	Approximation II (0 K)
La_2O_3	−3.170	−3.103 (0.067)	−3.705 (−0.535)
TiO	−2.351	−2.295 (0.056)	−2.797 (−0.446)
TiO_2 (anatase)	−2.670	−2.567 (0.103)	−3.236 (−0.566)
TiO_2 (rutile)	−2.683	−2.578 (0.105)	−3.247 (−0.564)
Ti_2O_3	−2.619	−2.534 (0.085)	−3.135 (−0.516)
Ti_3O_5	−2.631	−2.542 (0.089)	−3.169 (−0.538)

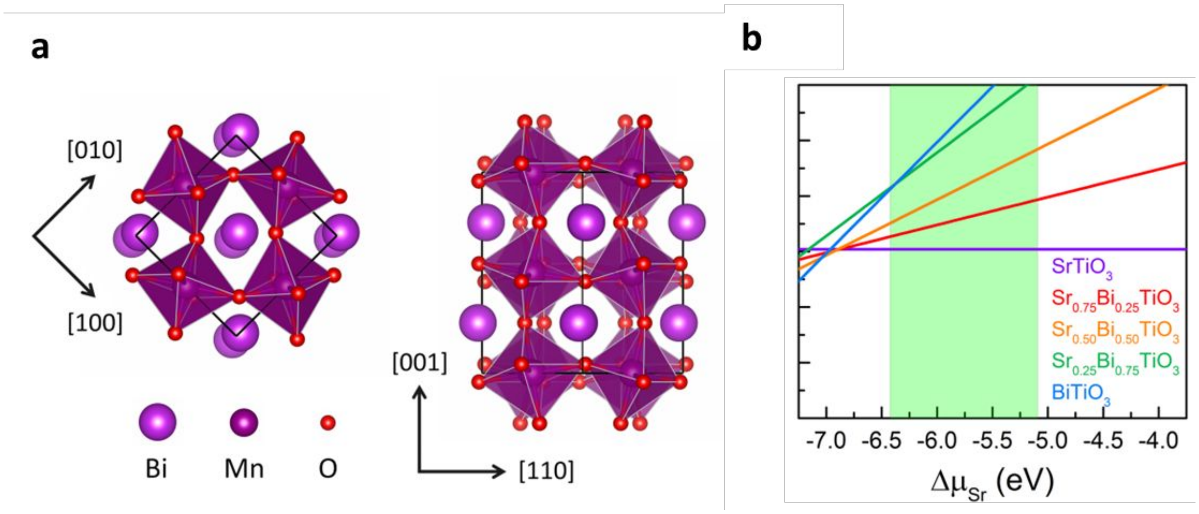

Figure S6. (a) The ground state structure of perovskite-type $BiMnO_3$ in the nanochannel, with space group *Pbnm*. (b) The reaction Gibbs free energy $\Delta_r G$ as a function of $\Delta\mu_{Sr}$ for $Sr_{1-x}Bi_xTiO_3$. The green shaded area represents the allowed chemical potential range as determined in Figure 5 (a).

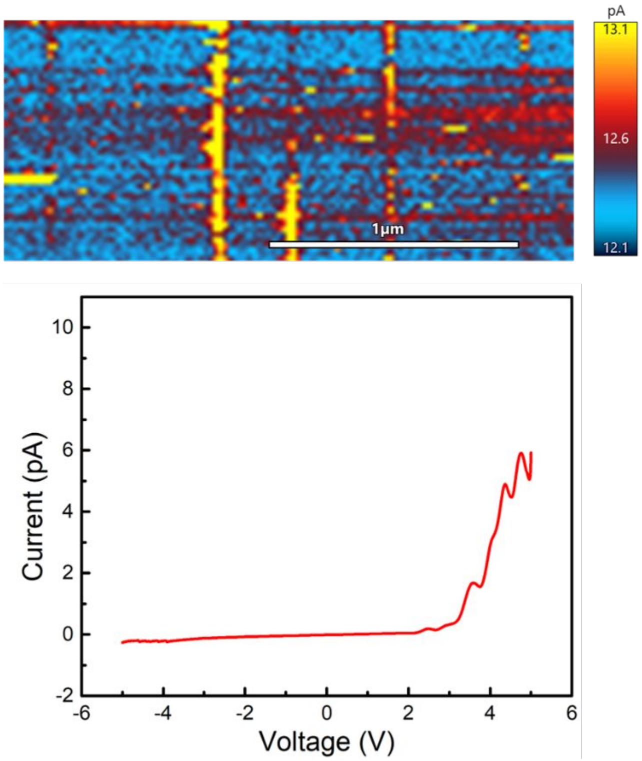

Figure S7. C-AFM conductivity measurement of the nanochannel using diamond-coated tip. Top: c-AFM conductivity mapping of the film surface. Bottom: I-V curves measured on the channel.

Methods of first-principles calculations

All density functional theory (DFT) calculations were performed using the projector augmented wave (PAW)[11, 12] method as implemented in the Vienna *ab* initio simulation package (VASP)[13, 14]. The exchange-correlation is described by the strongly constrained and appropriately normed (SCAN) functional[15], which has been shown to give overall improvement in reproducing the correct ground-state structures and accurate formation enthalpies[16-20]. The self-interaction error (SIE) is greatly reduced in the SCAN functional, but is not vanishing particularly when describing the highly correlated *d* electrons. To correct the SIE, an effective on-site Hubbard term[21] with a typical value of $U - J = 2$ eV for Ti[20, 22] and Mn[23] was introduced. An energy cutoff of 550 eV was used for the plane-wave basis expansion, and a Γ-centered Monkhorst-Pack *k*-mesh corresponding to 5000 per reciprocal atom was adopted for the Brillouin-zone integration. When calculating the SBH of LSMO/BMO junctions, we adopted the HSE06 hybrid functional[24] for bulk LSMO and BMO, so that we can get a more accurate description on the CBM and VBM at the cost of more computational resources.

Chemical potential of O. The chemical potential of O in the oxygen gas phase is related to experimental temperatures and pressures. Using the tabulated values for O_2 at standard states (enthalpy H_0 = 8.7 kJ·mol^{-1}, entropy S_O = 205 J·mol^{-1}·K^{-1} at T_0 = 298 K, P_0 = 1 bar), we express $\Delta\mu_O$ as:

$$\Delta\mu_O\left(T,P_0\right)=\frac{1}{2}\left[\left(H_0+\int_{T_0}^{T}C_p dT\right)-T\left(S_0+\int_{T_0}^{T}\frac{C_p}{T}dT\right)\right] \tag{3}$$

where $C_p = a + b\times10^{-3}T + c\times10^{6}T^{-2} + d\times10^{-6}T^{2}$ with *a*, *b*, *c* and *d* extracted from the thermodynamic tables[2] (a = 29.15, b = 6.48, c = –0.18, d = –1.02 in unit of kJ·mol^{-1}). At the

experimental conditions of $T = 650°C$ and $P = 1$ bar, we yielded $\Delta\mu_O = -1.00$ eV with respect to its ground state energy at 0 K.

Chemical potentials of La, Sr, Mn, Ti. The STO/LSMO films act as a reservoir for elements of La, Sr and Mn, restricting the allowed range of their chemical potentials. The stability condition of LSMO requires that:

$$0.75\Delta\mu_{La} + 0.25\Delta\mu_{Sr} + \Delta\mu_{Mn} + 3\Delta\mu_O = \Delta_f G(La_{0.75}Sr_{0.25}MnO_3) \tag{4}$$

where $\Delta_f G$ is the Gibbs free energy of formation per formula, approximated by the computed DFT formation energy as for all other solid phases. In LSMO there is neither precipitation of elemental solids, nor formation of competing binary and ternary phases, implying further constraints that:

$$\begin{aligned}
&\Delta\mu_{La} \le 0, \quad \Delta\mu_{Sr} \le 0, \quad \Delta\mu_{Mn} \le 0 \\
&2\Delta\mu_{La} + 3\Delta\mu_O \le \Delta_f G(La_2O_3) \\
&\Delta\mu_{Sr} + \Delta\mu_O \le \Delta_f G(SrO), \quad \Delta\mu_{Sr} + 2\Delta\mu_O \le \Delta_f G(SrO_2) \\
&\Delta\mu_{Mn} + \Delta\mu_O \le \Delta_f G(MnO), \quad \Delta\mu_{Mn} + 2\Delta\mu_O \le \Delta_f G(MnO_2) \\
&\Delta\mu_{La} + \Delta\mu_{Mn} + 3\Delta\mu_O \le \Delta_f G(LaMnO_3) \\
&\Delta\mu_{Sr} + \Delta\mu_{Mn} + 3\Delta\mu_O \le \Delta_f G(SrMnO_3)
\end{aligned} \tag{5}$$

where the spin configuration of each compound was set up according to the known experimental ground state. Equations (4) and (5) restrict the accessible range of $\Delta\mu_{La}$, $\Delta\mu_{Sr}$ and $\Delta\mu_{Mn}$ within the color filled polygon in Figure 4a. It can be clearly seen that the chemical potentials of La, Sr and Mn source from LSMO lie in a narrow range of $-8.66 \text{ eV} \le \Delta\mu_{La} \le -8.01$ eV, $-6.59 \text{ eV} \le \Delta\mu_{Sr} \le -5.42$ eV and $-4.73 \text{ eV} \le \Delta\mu_{Mn} \le -4.08$ eV. Similarly, we yielded $-6.42 \text{ eV} \le \Delta\mu_{Sr} \le -5.09$ eV and $-9.75 \text{ eV} \le \Delta\mu_{Ti} \le -8.42$ eV when STO acts as the reservoir.

TEM Imaging techniques

STEM HAADF imaging, EDS and EELS experiments were carried out on a JEOL Grand ARM300CF equipped with a cold field emission gun and double spherical aberration correctors with a spatial resolution of ~ 0.7 Å operating at 300 keV in Irvine Materials Research Institute at the University of California, Irvine. STEM images were taken with the convergence angle of the incident electrons at 32 mrad and the collection angle at 90-165 mrad. EDS mappings were acquired using dual silicon-drift detectors (SDDs). 50 scans (each with a 0.4 ms dwell time) in the same area across the interface were summed. The EELS spectrums were acquired with a dispersion of 0.25 eV/channel and a dwell time of 0.5 s/pixel. The pre-edge background in the EELS spectrums was removed by using power-law function in DigitalMicrograph. The in-plane conductive atomic force microscopy (c-AFM) were performed by using a commercial Asylum Research MFP-3D system.

Reference

1. Srilomsak, S., Thermal Expansion Studies on Cathode and Interconnect Oxides*. *ECS Proceedings Volumes* **1989,** *1989-11* (1), 129-140.
2. Uecker, R.; Velickov, B.; Klimm, D.; Bertram, R.; Bernhagen, M.; Rabe, M.; Albrecht, M.; Fornari, R.; Schlom, D. G., Properties of rare-earth scandate single crystals (Re=Nd−Dy). *Journal of Crystal Growth* **2008,** *310* (10), 2649-2658.
3. Muller, D. A.; Nakagawa, N.; Ohtomo, A.; Grazul, J. L.; Hwang, H. Y., Atomic-scale imaging of nanoengineered oxygen vacancy profiles in SrTiO3. *Nature* **2004,** *430* (7000), 657-661.
4. Atou, T.; Chiba, H.; Ohoyama, K.; Yamaguchi, Y.; Syono, Y., Structure Determination of Ferromagnetic Perovskite BiMnO3. *Journal of Solid State Chemistry* **1999,** *145* (2), 639-642.
5. Belik, A. A.; Iikubo, S.; Yokosawa, T.; Kodama, K.; Igawa, N.; Shamoto, S.; Azuma, M.; Takano, M.; Kimoto, K.; Matsui, Y.; Takayama-Muromachi, E., Origin of the Monoclinic-to-Monoclinic Phase Transition and Evidence for the Centrosymmetric Crystal Structure of BiMnO3. *Journal of the American Chemical Society* **2007,** *129* (4), 971-977.
6. Baettig, P.; Seshadri, R.; Spaldin, N. A., Anti-Polarity in Ideal BiMnO3. *Journal of the American Chemical Society* **2007,** *129* (32), 9854-9855.
7. Lee, J. H.; Ke, X.; Misra, R.; Ihlefeld, J. F.; Xu, X. S.; Mei, Z. G.; Heeg, T.; Roeckerath, M.; Schubert, J.; Liu, Z. K.; Musfeldt, J. L.; Schiffer, P.; Schlom, D. G., Adsorption-controlled growth of BiMnO3 films by molecular-beam epitaxy. *Appl Phys Lett* **2010,** *96* (26), 262905.
8. Hill, N. A.; Rabe, K. M., First-principles investigation of ferromagnetism and ferroelectricity in bismuth manganite. *Physical Review B* **1999,** *59* (13), 8759-8769.

9. Wagman, D. D.; E. W.; Parker, V. B.; Schumm, R. H.; Halow, I., The NBS tables of chemical thermodynamic properties: Selected values for inorganic and C1 and C2 organic substances in SI units. *National Institute of Standard and Technology* **1982,** 1, 405.

10. Binnewies M, M. E., Thermochemical data of elements and compounds. *Wiley Online Library* **2002**. DOI: 10.1002/9783527618347.

11. Blöchl, P. E., Projector augmented-wave method. *Physical Review B* **1994,** *50* (24), 17953-17979.

12. Kresse, G.; Joubert, D., From ultrasoft pseudopotentials to the projector augmented-wave method. *Physical Review B* **1999,** *59* (3), 1758-1775.

13. Kresse, G.; Furthmüller, J., Efficient iterative schemes for ab initio total-energy calculations using a plane-wave basis set. *Physical Review B* **1996,** *54* (16), 11169-11186.

14. Kresse, G.; Furthmüller, J., Efficiency of ab-initio total energy calculations for metals and semiconductors using a plane-wave basis set. *Computational Materials Science* **1996,** *6* (1), 15-50.

15. Sun, J.; Ruzsinszky, A.; Perdew, J. P., Strongly Constrained and Appropriately Normed Semilocal Density Functional. *Physical Review Letters* **2015,** *115* (3), 036402.

16. Zhang, Y.; Kitchaev, D. A.; Yang, J.; Chen, T.; Dacek, S. T.; Sarmiento-Pérez, R. A.; Marques, M. A. L.; Peng, H.; Ceder, G.; Perdew, J. P.; Sun, J., Efficient first-principles prediction of solid stability: Towards chemical accuracy. *npj Computational Materials* **2018,** *4* (1), 9.

17. Hinuma, Y.; Hayashi, H.; Kumagai, Y.; Tanaka, I.; Oba, F., Comparison of approximations in density functional theory calculations: Energetics and structure of binary oxides. *Physical Review B* **2017,** *96* (9), 094102.

18. Bartel, C. J.; Weimer, A. W.; Lany, S.; Musgrave, C. B.; Holder, A. M., The role of decomposition reactions in assessing first-principles predictions of solid stability. *npj Computational Materials* **2019,** *5* (1), 4.

19. Kitchaev, D. A.; Peng, H.; Liu, Y.; Sun, J.; Perdew, J. P.; Ceder, G., Energetics of MnO_2 polymorphs in density functional theory. *Physical Review B* **2016,** *93* (4), 045132.

20. Zhang, Y.; Furness, J. W.; Xiao, B.; Sun, J., Subtlety of TiO2 phase stability: Reliability of the density functional theory predictions and persistence of the self-interaction error. *The Journal of Chemical Physics* **2019,** *150* (1), 014105.

21. Dudarev, S. L.; Botton, G. A.; Savrasov, S. Y.; Humphreys, C. J.; Sutton, A. P., Electron-energy-loss spectra and the structural stability of nickel oxide: An LSDA+U study. *Physical Review B* **1998,** *57* (3), 1505-1509.

22. Long, O. Y.; Sai Gautam, G.; Carter, E. A., Evaluating optimal U for 3d transition-metal oxides within the SCAN+U framework. *Physical Review Materials* **2020,** *4* (4), 045401.

23. Sai Gautam, G.; Carter, E. A., Evaluating transition metal oxides within DFT-SCAN and SCAN+U frameworks for solar thermochemical applications. *Physical Review Materials* **2018,** *2* (9), 095401.

24. Krukau, A. V.; Vydrov, O. A.; Izmaylov, A. F.; Scuseria, G. E., Influence of the exchange screening parameter on the performance of screened hybrid functionals. *The Journal of Chemical Physics* **2006,** *125* (22), 224106.
